# Fine Mapping of the Leaf Rust Resistance Gene *Lr65* in Spelt Wheat ‘Altgold’

**DOI:** 10.3389/fpls.2021.666921

**Published:** 2021-06-28

**Authors:** Qiang Zhang, Wenxin Wei, Xiangxi Zuansun, Shengnan Zhang, Chen Wang, Nannan Liu, Lina Qiu, Weidong Wang, Weilong Guo, Jun Ma, Huiru Peng, Zhaorong Hu, Qixin Sun, Chaojie Xie

**Affiliations:** State Key Laboratory for Agrobiotechnology, Key Laboratory of Crop Heterosis and Utilization (MOE), Key Laboratory of Crop Genetic Improvement, College of Agronomy and Biotechnology, China Agricultural University, Beijing, China

**Keywords:** Altgold Rotkorn, *Lr65*, leaf rust resistance, fine mapping, marker-assisted selection

## Abstract

Wheat leaf rust (also known as brown rust), caused by the fungal pathogen *Puccinia triticina* Erikss. (Pt), is one by far the most troublesome wheat disease worldwide. The exploitation of resistance genes has long been considered as the most effective and sustainable method to control leaf rust in wheat production. Previously the leaf rust resistance gene *Lr65* has been mapped to the distal end of chromosome arm 2AS linked to molecular marker *Xbarc212*. In this study, *Lr65* was delimited to a 0.8 cM interval between flanking markers *Alt-64* and *AltID-11*, by employing two larger segregating populations obtained from crosses of the resistant parent Altgold Rotkorn (ARK) with the susceptible parents Xuezao and Chinese Spring (CS), respectively. 24 individuals from 622 F_2_ plants of crosses between ARK and CS were obtained that showed the recombination between *Lr65* gene and the flanking markers *Alt-64* and *AltID-11*. With the aid of the CS reference genome sequence (IWGSC RefSeq v1.0), one SSR marker was developed between the interval matched to the *Lr65*-flanking marker and a high-resolution genetic linkage map was constructed. The *Lr65* was finally located to a region corresponding to 60.11 Kb of the CS reference genome. The high-resolution genetic linkage map founded a solid foundation for the map-based cloning of *Lr65* and the co-segregating marker will facilitate the marker-assisted selection (MAS) of the target gene.

## Introduction

Virtually anywhere wheat is cultivated, its production is seriously constrained by fungal pathogens, and most significantly by single or multiple of the three species of rust (Hovmøller et al., 2010), i.e., leaf rust (*Puccinia triticina*); stem rust (*Puccinia graminis* f. sp. *tritici*); and stripe rust (*Puccinia striiformis* f. sp. *tritici*). Among these, leaf rust is considered potentially the most disruptive disease due to its more frequency and widespread occurrence in all wheat-growing locations of the world (Roelfs et al., 1992; Bolton et al., 2008; Huerta-Espino et al., 2011). Leaf rust can cause a 15% production reduction and heavy infection can lead to losses of up to 40% (Knott, 1989; McMullen et al., 2008). Over the past decades, outbreaks of rust diseases have occurred in various regions of China, resulting in severe wheat yield reduction (Chen et al., 2018). Although leaf rust can be controlled through foliar fungicide applications, the most effective and eco-friendly way to control the disease is based on improved varieties containing resistance genes (Keller et al., 2008). However, one of the most frustrating issues in disease resistance breeding is the failure of resistance genes, due to the evolving nature of plant pathogens resulting in new virulent races that can cause disease in formerly resistant wheat varieties. Therefore, it is necessary to search for new diverse effective resistance genes that can be used in wheat breeding programs.

To date, more than 80 leaf rust resistance genes (*Lr*) have been identified (Singh et al., 2013; Qureshi et al., 2018; Kumar et al., 2021). Roughly half of these genes are from wild relatives of wheat, while the remainder are from cultivated wheat (Marais et al., 2005; Naik et al., 2015; Rani et al., 2020). Wild relatives of wheat provide a huge gene pool of agronomy utility, including genes for rust resistance (Narang et al., 2019). The D genome donor of wheat, *Aegilops tauschii*, has been a rich source of resistance genes (Gill et al., 2019). Leaf rust resistance genes *Lr21*, *Lr32*, and *Lr39* have been transferred from *Ae. tauschii* into bread wheat (Raupp et al., 2001; Huang et al., 2003; Thomas et al., 2010). Tetraploid wheat is another important origin of disease resistance (Singh et al., 2017). *Lr14a*, *Lr23*, and *Lr53* were derived from durum wheat or wild emmer wheat and were used in common wheat breeding (McIntosh et al., 1995; Marais et al., 2005).

Spelt wheat (*Triticum spelta*) is an ancient crop that has been cultivated since 5000 BC (Xie et al., 2015). It is still a minor crop used for bread and fodder in Europe and North America today (Campbell, 1997). Spelt wheat has the same AABBDD genome as common wheat and their hybrids are fertile, facilitating the transfer of desirable genes to common wheat. In addition to genetic variation in protein concentration (Gomez-Becerra et al., 2010), lipid and mineral nutrient contents (Ruibal-Mendieta et al., 2002; Zhao et al., 2009), spelt wheat also shows excellent resistance to wheat rusts. Examples are the wheat yellow rust resistance gene *Yr5* gene, derived from spelt and localized on the long arm of chromosome 2B (Sun et al., 2002; Yan et al., 2003), and the *Lr44* gene in the Spelt variety 7831, located chromosome 1B (Dyck and Sykes, 1994).

Molecular markers have been used extensively in wheat breeding, principally for genetic mapping, marker-assisted selection (MAS), and positional gene cloning (Jost et al., 2020). With the evolution of sequencing technology, marker development has shifted to the sequencing era (Paux et al., 2012). The release of the annotated genome sequence of Chinese Spring (CS) have greatly improved our understanding of the wheat genome and facilitated the efficiency of marker development in wheat (Li et al., 2020).

The spelt wheat Altgold Rotkorn (ARK), a Swiss variety (Pedigree: Oberkulmer/Sandmeier), was first released in 1952. Wang et al. (2010) identified a leaf rust resistance gene *LrAlt* in Altgold and localized it to the distal end of the short arm of chromosome 2A. Mohler et al. (2012) reported the characterization and mapping of the same leaf rust resistance gene *LrARK0;* in ARK. Since *LrAlt* and *LrARK0;* were from the same germplasm and located at the same position, they were designated as *Lr65* (Mohler et al., 2012). In this study, we performed fine mapping of *Lr65* gene by exploring the CS reference genome. Our analysis located *Lr65* gene to a 60.11 Kb region on the IWGSC Ref-Seq v1.0 and identified one most likely candidate gene for *Lr65* in Altgold by comparing genome resequencing data between resistant and susceptible parents. In addition, co-segregating molecular markers were developed for MAS of the target gene.

## Materials and Methods

### Plant and Pathogen Materials

Altgold, a spelt wheat cultivar with high resistance to leaf rust, was crossed with two susceptible common wheat lines “Xuezao” and “CS”, and two F_2_ segregating populations (Xuezao/Altgold and CS/Altgold) were constructed. These two populations were used for the genetic analysis and mapping of leaf rust resistance gene. In all experiments, a susceptible common wheat line of Xuezao was used as a comparison to check for successful inoculation. The *P. triticina* isolate PHT (provided by Institute of Plant Protection, Chinese Academy of Agricultural Sciences, Beijing, China) was used for the inoculation. PHT was avirulent on Altgold and virulent on Xuezao and CS. The conidia were propagated in the greenhouse on the susceptible plants.

### Plant Growth and Pathogen Infection

The parental plants of Altgold, Xuezao and CS and F_2_ populations were tested for leaf rust resistance at seedling stage. The inoculations were initiated when the first leaves were fully unfolded, by spraying 1% Tween-20 aqueous solution as surfactant and then brushing conidia from the susceptible seedlings with sporulating leaf rusts onto the seedlings to be tested. The inoculated seedlings were incubated in dark plastic-covered boxes for 48 h at 15°C and 100% relative humidity and then transferred to greenhouse. 10–14 days after inoculation, infection types (ITs) were scored on a scale of 0–4 (0 = hypersensitive flecks, 1 = small uredinia with necrosis, 2 = moderate size pustules with chlorosis, 3 = moderate-large size uredinia without necrosis or chlorosis, and 4 = large uredinia lacking necrosis or chlorosis) (Stakman et al., 1962). ITs 0–2 represent resistance and ITs 3–4 represent susceptibility.

### DNA Extraction and Quantification

DNA was extracted from seedlings of the F_2_ populations and parents Altgold (resistant parent) as well as Xuezao and CS (susceptible parents) using the CTAB method (Maroof et al., 1994). DNA samples were quantified using a NanoDrop One spectrophotometer instrument (Nanodrop Technologies) and diluted to a concentration of 30 ng/μl.

### Resequencing of Resistant Parent Altgold

To obtain genomic variations between Altgold and CS, we performed whole-genome resequencing of Altgold. Altgold’s whole genome sequencing was performed using the Illumina HiSeq2500 sequencing platform for double-end sequencing. The library construction and sequencing were performed by Beijing Novogene company. The read length of the paired-end sequencing library was 150 bp, the raw sequencing data were processed according to GATK’s best practices workflow (Van der Auwera et al., 2013).

### Molecular Marker Development

*Lr65* gene had already been mapped distal to marker *Xbarc212* on chromosome arm 2AS (Wang et al., 2010). Simple sequence repeats (SSRs) were developed based on the CS reference genome sequence distal to the *Xbarc212* locus. Meanwhile, InDels with insert/deletion size > 3bp were selected from the target interval between Altgold re-sequencing and CS reference genome sequence alignment database for further marker design. InDel polymerase chain reaction (PCR) primers were designed using Primer3Plus^1^, with amplicon sizes ranging from 100 to 500 bp. BatchPrimer3 v1.0^2^ was used to develop SSR markers.

### Polymerase Chain Reaction Amplification and Visualization

Polymerase chain reaction amplification was performed in a 10 μL reaction volume containing 6 μL of 2 × Tag PCR StarMix with loading dye, 35–120 ng/mL DNA 2 μL, 1 μL of primer (mix of forward and reverse primers, 2 mM) and 1 μL of ddH_2_O. The thermal profile consists of an initial denaturation step at 94°C for 5 min, followed by 35 cycles of 94°C for 30 s (denaturation), 50–61°C (depending on the annealing temperature of the specific primer) for 30 s, 72°C for 30 s (primer extension), and a terminal extension at 72°C for 10 min, stored at 4°C. The PCR products were separated by 10% non-denaturing polyacrylamide gel electrophoresis (acrylamide: bisacrylamide = 39:1), and gels were visualized with silver nitrate staining (Bassam et al., 1991).

### Linkage Analysis and Map Construction

A chi-square analysis was performed on the leaf rust test data to confirm the goodness of fit of the observed ratios from the F_2_ populations to the theoretical expected values. The χ^2^ analysis was executed in Microsoft Excel (version 2010) using the Bchitest^ function to calculate χ^2^ and *p*-values. The polymorphic markers tested between resistant and susceptible parents were used to genotype 2144 F_2_ plants. The phenotypic data of disease responses were used for linkage analysis in combination with PCR amplification results. The localization of markers and the target gene is fulfilled based on recombination between markers genotype data and resistance/susceptibility phenotype data. Genetic distances were calculated in centiMorgan (cM).

### Physical Mapping and Gene Annotation

The sequences of the two closest flanking markers linked to *Lr65* were used as lookups for a searches of the IWGSC RefSeq v1.0 to define the physical interval covering *Lr65* locus on CS chromosome 2AS. The gene annotation for the target interval was retrieved from the IWGSC RefSeq v1.0 annotation^3^.

### Genomic Comparison Among Multiple Wheat Varieties

The sequence information was obtained from the Triticeae Multi-omics Center^4^ to obtain sequence information of annotated genes in candidate intervals, and then using the wheat 10 + genome^5^ (Walkowiak et al., 2020) for sequence alignment between the genomes of 15 wheat varieties.

## Results

### Genetic Analysis of the Leaf Rust Resistance Gene *Lr65* in Two Segregating Populations

**FIGURE 1:**
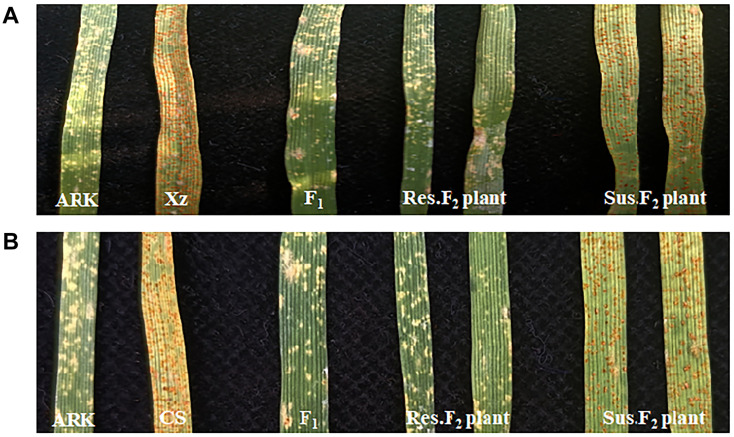
Phenotype of seedling responses after inoculation with *Pt* race PHT. **(A)** Altgold (ARK), Xuezao (Xz), F_1_ (Xz/Altgold), and typical resistant and susceptible F_2_ individuals. **(B)** Altgold (ARK), CS, F_1_ (CS/Altgold) and resistant and susceptible F_2_ of individuals.

At the seedling stage, the parental lines Xuezao and CS demonstrated a clear susceptible response to the leaf rust isolate PHT with an infection type (IT) score of 3, while Altgold showed a high-level resistant response with an IT score of 0 (Figures 1A,B). The F_1_ plants and F_2_ populations of Xuezao/Altgold and CS/Altgold were examined for the responses to the inoculation of the Pt isolate PHT at the seedling stage as well, along with the parents. The F_1_ plants showed the same approximate immune infection type as the resistant parent Altgold, indicating the complete dominance of the resistance (Figures 1A,B). Of the 1522 F_2_ plants screened from the Xuezao/Altgold cross, 1130 were resistant and 392 susceptible, fitting the ratio of 3:1 (χ^2^_3:1_ = 0.46, *p* > 0.05). In the F_2_ population derived from cross CS/Altgold, 454 plants were resistant and 168 susceptible (χ^2^_3:1_ = 1.33, *p* > 0.05). The segregation of these two populations confirm that the leaf rust resistance in Altgold is controlled by a single dominant gene (Table 1), which is most likely the gene *Lr65* (previously known as *LrAlt* or *LrARK0*) (Wang et al., 2010; Mohler et al., 2012).

**TABLE 1:** Segregation for leaf rust resistance in the Xuezao/Altgold and CS/Altgold F_2_ population.

		Number of seedling plants			
		
Cross	Population	Resistant	Susceptible	Total	χ ^2^_(3:1)_
Xuezao/Altgold	F_2_	1130	392	1522	χ^2^ = 0.46, *p* = 0.49
CS/Altgold	F_2_	454	168	622	χ^2^ = 1.33, *p* = 0.24

### Marker Discovery and Molecular Mapping

**FIGURE 2:**
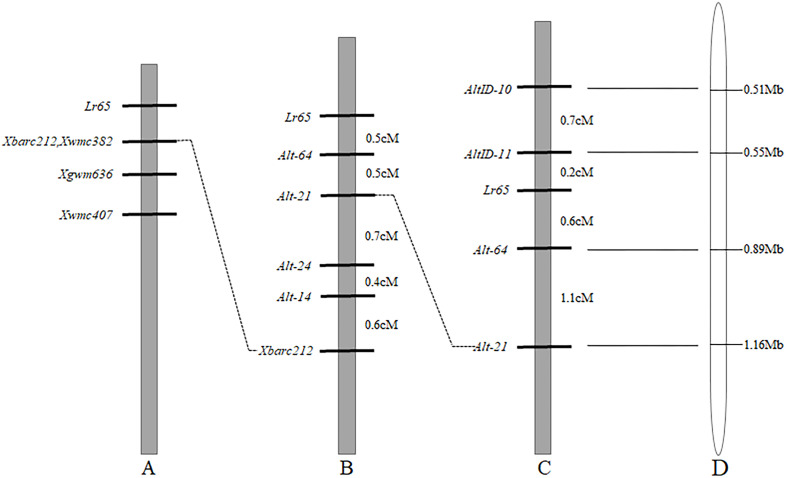
Comparison of genetic linkage maps of *Lr65* on chromosome 2AS and the corresponding physical location on Chinese Spring RefSeq v1.0. **(A)** Previous map of Wang et al. (2010). **(B)** The map of *Lr65* in current study based on Xuezao/Altgold F_2_ population, genetic distances were indicated in cM on the right-hand side. **(C)** The map of *Lr65* in current study based on CS/Altgold F_2_ population, genetic distances are indicated in cM on the right-hand side. **(D)** The physical location of markers of *Lr65* on the chromosome 2AS of Chinese Spring RefSeq v1.0. The physical distances were shown on the right in Mb.

Since *Lr65* gene has been located on the terminus of the short arm of chromosome 2A and the closest marker to *Lr65* is *Xbarc212* (Figure 2A) (Wang et al., 2010; Mohler et al., 2012). To further increase the map resolution in the *Lr65* region, new markers were developed using various genomic resources. 88 SSR primers were developed based on CS chromosome 2AS reference genome sequence (RefSeq v1.0) and tested on the two parents (Altgold and Xuezao). Four polymorphic markers (*Alt-14*, *Alt-21*, *Alt-24*, and *Alt-64*) were identified (Table 2). A total of 1522 Xuezao/Altgold F_2_ plants were genotyped with these four markers, and 47 plants were identified with recombination between the marker loci and the resistance gene. Linkage analysis indicated that the closest marker to *Lr65* was *Alt-64* with a genetic distance of 0.5 cM. All four markers were on the proximal side to *Lr65* and closer than *Xbarc212* (Figure 2B).

**TABLE 2:** The primer sequences used in this study.

Marker	Forward primer (5′–3′)	Reverse primer (5′–3′)	Marker type	Product size (bp)	Physical position (bp)	
					
					Start	End
*AltID-10*	CATCACTTTTGTCTCATCCA	CTATAACCCTGGCCCTTTAATA	Indel	153	517281	517434
*AltID-11*	AGAGGCTATGGATTGGAGTAG	CGCCATTAATGTCCATATCA	Indel	249	555551	555800
*Alt-92*	GTCCCTCTACAGTTCCATCC	GTGAAAACCATGTTGCAAAG	SSR	206	615668	615874
*Alt-64*	AATCACATCACCCGACTCT	CGATTTCTACCTTTCTGGACT	SSR	173	891823	891996
*Alt-21*	GTAAAATAGAGGAGGGGTGAA	CATGTTAGAAGGGATAGAGAGG	SSR	144	1166351	1166495
*Alt-24*	ACCCAATGCACTTGTACTCTAT	CTGGTGAATGGATGAAACA	SSR	135	1227798	1227933
*Alt-14*	GCGAACAGAAAGAAAGAAAG	CCTAGACAGCACACATCTTGTA	SSR	152	1309861	1310013
*1500-1*	ATTCCATTGCCGGTCTATCTT	GCACCTCCTTTTTGTTGTTG	Indel	108	583323	583431
*Xbarc-212*^a^	GGCAACTGGAGTGATATAAATACCG	CAGGAAGGGAGGAGAACAGAGG	SSR	185	1582751	1582936

*^*a*^The marker *Xbarc212* was used in a previous study (Wang et al., 2010).*

To obtain markers on the other side of *Lr65*, we compared the resequencing data of Altgold with the CS reference in the target region, which corresponds to the most distal 1.16 Mb interval of chromosome 2AS in CS RefSeq v1.0. Based on the Indel variations between the two parents, we designed eighteen Indel markers, two (*AltID-10* and *AltID-11*) of which were tested polymorphic between the parents (Table 2). These two Indel markers and previously developed SSR markers (*Alt-21* and *Alt-64*) were used to genotype 622 F_2_ plants of the cross CS/Altgold. A genetic linkage map spanning 2.6 cM was constructed using these four markers (Figure 2C). In this map, *Lr65* gene is delimited to a genetic interval of 0.8 cM, flanked by markers *Alt-64* and *AltID-11*, with *AltID-11* 0.2cM distal to *Lr65* and *Alt-64* 0.6cM to *Lr65* on the proximal side.

**FIGURE 3:**
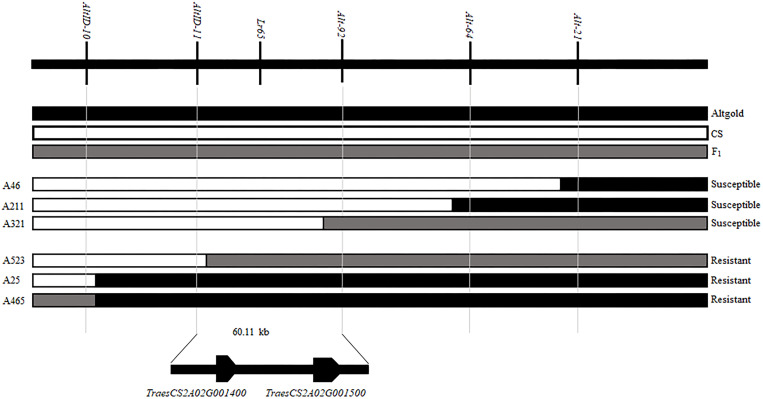
Fine mapping of *Lr65* and two annotated genes in the target interval on the Chinese spring reference genome. The phenotypes and genotypes of six F_2_ recombinants are displayed. The code and phenotype of each individual were put on the left and right sides, respectively. Black, white and gray blocks represent the genomic regions of Altgold, CS, and heterozygotes, respectively.

When we matched the sequences of *Alt-64* and *AltID-11* with the genome sequence of CS (IWGSC v1.0), we found that the two markers were spanning an area of about 0.34 Mb (555551–891823) on CS chromosome 2AS (Figure 2D). Based on Altgold’s re-sequencing data matching this 0.34 Mb interval, 11 SSR primers were designed and one more polymorphic marker *Alt-92* was found between Altgold and CS (Table 1). After tested among the 24 recombinants previously obtained by screening with the flanking markers *Alt-21*, *Alt-64* and *AltID-10*, *AltID-11*, two recombinants were identified between *Alt-92* and *Lr65*. These results showed that the *Lr65* locus was located between the markers *AltID-11* and *Alt-92* (Figure 3).

### Physical Mapping and Gene Annotation of the *Lr65* Target Interval

**FIGURE 4:**
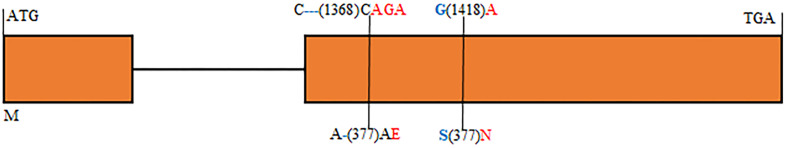
Structure of the annotated gene *TraesCS2A02G001500* displaying nucleotide and amino acid sequence polymorphisms between the resistant and susceptible parents. Introns and exons are indicated by lines and orange boxes, respectively. Blue and red color characters indicate alleles of resistant and susceptible parents, respectively. Numbers in parentheses represent the positions of nucleotide and amino acid sequences relative to ATG and M. – Indicates a sequence deletion.

In order to physically locate *Lr65*-linking markers, the sequences of all markers which were anchored in the high-resolution gene map were aligned to the CS reference genome sequence. The relative physical positions of these markers were generally consistent with the genetic linkage map (Figure 2). The closest flanking markers *AltID-11* and *Alt-92* of *Lr65* delimitated a 60.11 Kb (555,551–615,668) interval in the CS Reference Genome (RefSeq v1.0). This region encompasses two annotated protein-coding genes, *TraesCS2A02G001400* and *TraesCS2A02G001500*, according to the IWGSC RefSeq v1.0 annotation^6^ (see text foot note 3) (Figure 3). The two annotated genes were put on NCBI^7^ to predict their protein structures, we found that *TraesCS2A02G001400* encodes a protein similar to that found in intracellular human pathogens with a conserved regions of internalin_A super family and *TraesCS2A02G001500* encodes a typical disease resistance protein (R protein) with a NB-ARC domain at the N-terminal end and three contiguous LRR at the C-terminal end (Supplementary Figure 1 and Table 3). One 3 bp Indel and one SNP were found in the coding sequence of *TraesCS2A02G001500* (Figure 4 and Supplementary Figure 3), indicating that these differences may lead to different protein functions, while there is no difference in sequence of *TraesCS2A02G001400* between Altgold and CS (Supplementary Figure 2). Therefore, *TraesCS2A02G001500* is most likely the candidate gene of *Lr65*.

**TABLE 3:** Candidate genes in the most distal 60.11kb region of 2AS.

No.	Gene ID	Start position of the gene^a^	Length of gene (bp)	Gene annotation	Conservative regions
1	TraesCS2A02G001400	562911	2915	found in the intracellular human pathogen	internalin_A super family
2	TraesCS2A02G001500	580888	4532	Disease resistance protein	NB-ARC and LRR

*^*a*^Gene ID and positions were fetched from the URGI website (https://urgi.versailles.inra.fr/) and as per IWGSC gene annotation v1.0.*

### Comparison Among the Genomes of Multiple Wheat Cultivars

**FIGURE 5:**
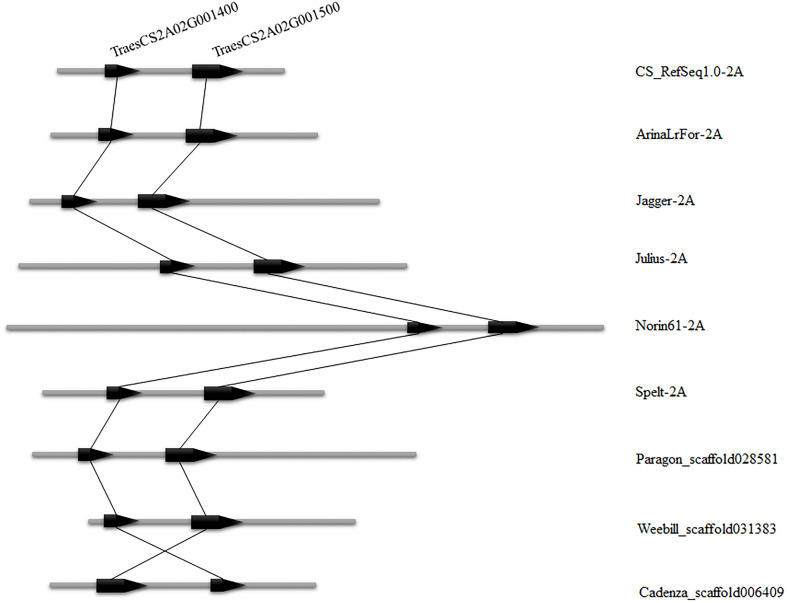
Candidate interval of two genes *TraesCS2A02G001400* and *TraesCS2A02G001500* compared among nine wheat varieties. The left side indicates the position of the gene on the chromosome and the right side is the wheat variety name.

To validate the consistency of collinearity within candidate intervals in multiple wheat varieties, the wheat 10 + genome^8^ (Walkowiak et al., 2020) was used for comparison between genomes of additional wheat materials. Six wheat varieties (CS_RefSeq1.0, ArinaLrFor, Jagger, Julius, Norin61, Spelt) were identified in which both genes in the candidate interval were matched to chromosome arm 2AS, while three varieties were found matched to the same scaffold (Paragon_scaffold, Weebill_ scaffold, and Cadenza_scaffold). The number of genes in these nine wheat varieties was consistent within the candidate interval, and the order of these two genes in these varieties was the same as in CS, with only one reversed (Cadenza_scaffold) (Figure 5). This indicates that the number of genes in the candidate region is uniform in multiple wheat varieties.

### Development of the Diagnostic Marker of *Lr65*

**FIGURE 6:**
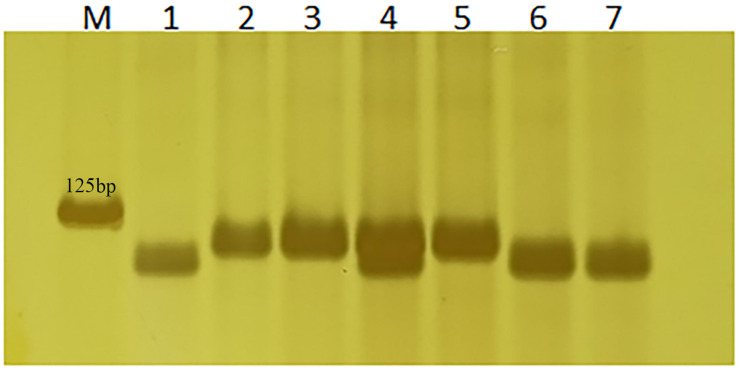
Verification of *Lr65*’s diagnostic marker 1500-1. Nos. 1–3 are Altgold, Xuezao and CS. Nos. 4–7 are recombinants A523, A25, A321, and A211, respectively. M is marker.

Based on the 3-bp Indel in *TraesCS2A02G001500* between Altgold and CS, marker *1500-1* was developed and validated on Altgold, Xuezao, and CS and the key recombinants (A25, A211, A321, and A523) (Table 2 and Figure 6). The test result indicated marker *1500-1* was co-segregating with *Lr65* gene (Figure 3).

**FIGURE 7:**
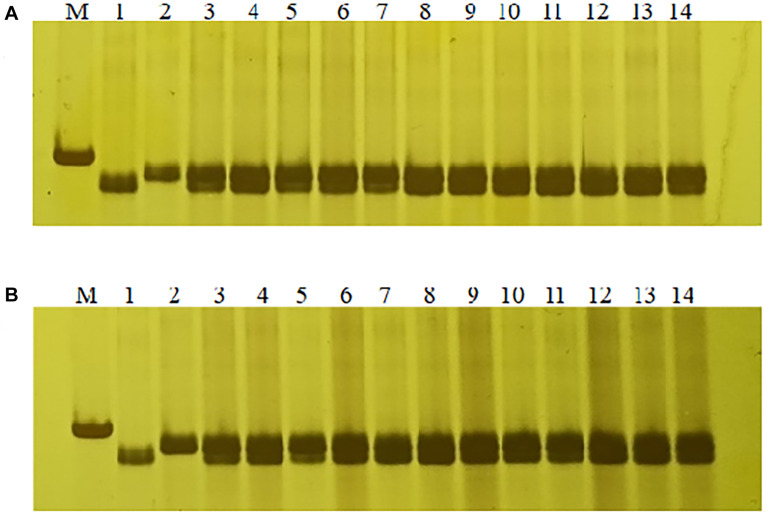
Marker-assisted selection of *Lr65* with diagnostic marker 1500-1. **(A)** Selection of *Lr65* in “Xuexao/Altgold//Shiyou 20” population. 1: a resistant progeny of Xuexao/Altgold, 2: Shiyou20, 3–14: the resistant plants among the progenies. **(B)** Selection of *Lr65* in “Xuexao/Altgold//Zhongmai 1062” population. 1: a resistant progeny of Xuexao/Altgold, 2: Zhongmai1062, 3–14: the resistant plants among the progenies. M is marker.

In order to confirm the usefulness of this *Lr65* co-segregating marker in breeding, we tested marker *1500-1* on other 18 different Chinese wheat cultivars, we found that the PCR product size of marker *1500-1* in Altgold containing *Lr65* was unique and not detected in the other cultivars (Supplementary Figure 4); therefore, marker *1500-1* is diagnostic for selection of *Lr65* gene. Then we screened the marker *1500-1* in two other populations of F_1_ progenies of crosses “Xuexao/Altgold//Shiyou 20” and “Xuexao/Altgold//Zhongmai 1062” and found that the marker was 100% associated with the leaf rust resistance (Figure 7 and Supplementary Figure 4). Since the resistant plants were the results of combining of *Lr65* with the susceptible alleles of Shiyou 20 and Zhongmai 1062, these plants all showed the heterozygous banding of marker *1500-1*.

## Discussion

In addition to *Lr65*, four wheat leaf rust resistance genes was located on the short arm of chromosome 2A, including *Lr17*, *Lr37*, and *Lr45* (McIntosh et al., 2008; Prasad et al., 2020). *Lr11* was previously located on chromosome 2AS (Soliman et al., 1964), but recent studies have shown that *Lr11* is located distal to chromosome 2DS (Darino et al., 2015). The gene *Lr17* has two resistance alleles, *Lr17a* and *Lr17b* (Dyck and Kerber, 1977; Singh et al., 2001). *Lr17a* was flanked by marker *Xgwm614* (distal) and *Xgwm407* (proximal), while marker *Xgwm636* was distal to *Xgwm614* (Bremenkamp-Barrett et al., 2008). *Lr65* (*LrAlt*) is mapped distal to *Xgwm636* (Wang et al., 2010). Gene *Lr37* is located within a fragment of *Ae. ventricosa* (Tausch) Cess. chromosome 2NS translocated to bread wheat chromosome 2AS, and genetic mapping analysis showed that the 2NS translocation replaced about half of the short arm of chromosome 2A (Helguera et al., 2003). The gene *Lr45* is from rye chromosome 2R translocated to wheat chromosome 2A (Zhang et al., 2006). According to the above information, we conclude that *Lr65* is a unique leaf rust resistance gene.

Previously *Lr65* was mapped distal to the closest marker *Xbarc212* on wheat chromosome 2AS (Wang et al., 2010; Mohler et al., 2012). In this study, using two large F_2_ segregating populations of crosses Xuezao/Altgold and CS/Altgold, we fine mapped *Lr65* and narrow down it between markers AltID-11 and Alt-92, corresponding to the 60.11 Kb (555,551–615,668) interval according to the CS Reference Genome.

The gene fine mapping involved developing more polymorphic markers covering the genetic interval of the target gene. With the release of whole genome Reference sequence of CS, development of polymorphic markers associated with a target gene is becoming easier. The process of fine mapping of *Lr65* illustrates the effectiveness of the reference genome information and the resequencing data of the specific parental lines for the guided development of markers to target genes. Our work also demonstrate the advantage of using different crosses in the genetic mapping. Even though additional closer markers to *Lr65* were found using the Xuezao/Altgold F_2_ population, all were on one side to the target gene. When we changed to the CS/Altgold population, the target gene was successfully delimitated by flanking markers and narrow down to a shorter interval.

In the 60.11-Kb interval that contains *Lr65* locus on CS 2AS, there are two protein-coding genes annotated, *TraesCS2A02G001400* and *TraesCS2A02G001500*, according to the IWGSC RefSeq v1.0 annotation (see text foot note 3). Sequence analysis showed no difference in *TraesCS2A02G001400* between the resistant and susceptible parents (Altgold, CS and Xuezao). However, we found two sequence variations (one 3-bp Indel and one SNP) between Altgold and CS in the coding region of *TraesCS2A02G001500*. One marker was developed to tag the 3-bp Indel variation between the parents and found to be co-segregating with *Lr65*. *TraesCS2A02G001500* was predicted to encode a protein with nucleotide binding sites and multiple leucine-rich repeats (NBS-LRR), the typical structures of disease resistance genes (R genes). Many cloned wheat rusts resistance genes are found to encode NBS-LRR proteins, including leaf rust resistance genes (*Lr1*, *Lr10*, *Lr21*, and *Lr22*) (Feuillet et al., 2003; Huang et al., 2003; Hiebert et al., 2007; Qiu et al., 2007), stripe rust resistance genes (*Yr5* and *Yr10*) (McGrann et al., 2014; Yuan et al., 2018), and stem rust resistance genes (*Sr22*, *Sr33*, *Sr35*, *Sr45*, and *Sr50*) (Saintenac et al., 2013; Periyannan et al., 2014; Casey et al., 2016; Saur et al., 2019; Md Hatta et al., 2020). Our results suggest that *TraesCS2A02G001500* might be the candidate gene of *Lr65*. The works to verify the disease resistance function of the Altgold allele of *TraesCS2A02G001500* are underway. However, there is still a chance that the sequence corresponding to *Lr65* is absent in CS genomic sequence. If so, we need to construct a genomic library of Altgold and to clone *Lr65* by physical mapping of contigs. The closest flanking and co-segregating markers developed in our present study will greatly aid the map-based cloning of *Lr65*.

Spelt is genetically distant from common wheat and with a high degree of genetic variation unexploited (Würschum et al., 2017; Akel et al., 2018). *Lr65* was first identified in spelt wheat and not being widely used in common wheat breeding. In addition to the resistance to the Chinese isolate PHT as in this study, *Lr65* was resistant to many Australia and Germany *P. triticina* isolates (Mohler et al., 2012). Utilization of *Lr65* will help to diversify the resistance genes in common wheat breeding and help to protect wheat production. However, due to the evolution of new virulent pathogen isolates, major disease resistance genes are prone to lose their effectiveness when deployed alone. Mohler et al. (2012) had reported the existence of virulent pathotypes for *Lr65*. The *Lr65* gene was recommended to be used in combination with other resistance genes for the protection against leaf rust. The co-segregating marker we developed in present study would be helpful to pyramid *Lr65* with other resistance genes.

## Data Availability Statement

The datasets for this study can be found in the EVA repository (a preview of the data can be viewed here: https://wwwdev.ebi.ac.uk/eva/?eva-study=PRJEB45547). Project: PRJEB45547 and Analyses: ERZ2470822. SS ID: ss7173984454 and ss7173984455.

## Author Contributions

CX designed the research. QZ and CX conducted the research. CX, QZ, and WXW prepared the samples. QZ, XZ, SZ, CW, and NL analyzed the data. QZ wrote the draft. NL, LQ, CX, WG, JM, HP, ZH, and QS made the revision of the manuscript. All authors read and approved the manuscript.

## Conflict of Interest

The authors declare that the research was conducted in the absence of any commercial or financial relationships that could be construed as a potential conflict of interest.

## Footnotes

1 http://www.bioinformatics.nl/cgi-bin/primer3plus/primer3plus.cgi

2 https://probes.pw.usda.gov/batchprimer3/

3 http://urgi.versailles.inra.fr/jbrowseiwgsc/gmod_jbrowse/

4 http://202.194.139.32/getfasta/index.html

5 https://webblast.ipk-gatersleben.de/wheat_ten_genomes/

6 http://ncbi.nlm.nih.gov/Structure/cdd/wrpsb.cgi

7 https://webblast.ipk-gatersleben.de/wheat_ten_genomes/

## References

[B1] Akel W. Thorwarth P. Mirdita V. Weissman E. A. Liu G. Würschum T. (2018). Can spelt wheat be used as heterotic group for hybrid wheat breeding? *Theor. Appl. Genet.* 131 973–984. 10.1007/s00122-018-3052-3 29340753

[B2] Bassam B. J. Caetano-Anollés G. Gresshoff P. M. (1991). Fast and sensitive silver staining of DNA in polyacrylamide gels. *Anal. Biochem.* 196 80–83. 10.1016/0003-2697(91)90120-i 1716076

[B3] Bolton M. D. Kolmer J. A. Garvin D. F. (2008). Wheat leaf rust caused by *Puccinia triticina*. *Mol. Plant Pathol.* 9 563–575. 10.1111/j.13643703.2008.00487.x 19018988 PMC6640346

[B4] Bremenkamp-Barrett B. Faris J. D. Fellers J. P. (2008). Molecular mapping of the leaf rust resistance gene Lr17a in wheat. *Crop Sci.* 48 1124–1128.

[B5] Campbell K. G. (1997). Spelt: agronomy, genetics, and breeding. *Plant Breed. Rev.* 15 187–214. 10.1002/9780470650097.ch6

[B6] Casey L. W. Lavrencic P. Bentham A. R. Cesari S. Ericsson D. J. Croll T. (2016). The CC domain structure from the wheat stem rust resistance protein Sr33 challenges paradigms for dimerization in plant NLR proteins. *Proc. Natl. Acad. Sci. U. S. A.* 113 12856–12861. 10.1073/pnas.1609922113 27791121 PMC5111715

[B7] Chen D. Shi Y. Huang W. Zhang J. Wu K. (2018). Mapping wheat rust based on high spatial resolution satellite imagery. *Comput. Electron. Agric.* 152 109–116. 10.1016/j.compag.2018.07.002

[B8] Darino M. A. Dieguez M. J. Singh D. Ingala L. R. Pergolesi M. F. Park R. F. (2015). Detection and location of Lr11 and other leaf rust resistance genes in the durably resistant wheat cultivar Buck Poncho. *Euphytica* 206 135–147.

[B9] Dyck P. L. Kerber E. R. (1977). Inheritance of leaf rust resistance in wheat cultivars Rafaela and EAP 26127 and chromosome location of gene Lr17. *Can. J. Genet. Cytol.* 19 355–358.

[B10] Dyck P. L. Sykes E. E. (1994). Genetics of leaf-rust resistance in three spelt wheats. *Can. J. Plant Sci.* 74 231–233. 10.4141/cjps94-047

[B11] Feuillet C. Travella S. Stein N. Albar L. Nublat A. Keller B. (2003). Map-based isolation of the leaf rust disease resistance gene Lr10 from the hexaploid wheat (Triticum aestivum L.) genome. *Proc. Natl. Acad. Sci. U. S. A.* 100 15253–15258. 10.1073/pnas.2435133100 14645721 PMC299976

[B12] Gill H. S. Li C. Sidhu J. S. Liu W. Wilson D. Bai G. (2019). Fine mapping of the wheat leaf rust resistance gene Lr42. *Int. J. Mol. Sci.* 20:2445. 10.3390/ijms20102445 31108903 PMC6567072

[B13] Gomez-Becerra H. F. Erdem H. Yazici A. Tutus Y. Torun B. Ozturk L. (2010). Grain concentrations of protein and mineral nutrients in a large collection of spelt wheat grown under different environments. *J. Cereal Sci.* 52 342–349. 10.1016/j.jcs.2010.05.003

[B14] Helguera M. Khan I. A. Kolmer J. Lijavetzky D. Zhong-Qi L. Dubcovsky J. (2003). PCR assays for the Lr37-Yr17-Sr38 cluster of rust resistance genes and their use to develop isogenic hard red spring wheat lines. *Crop Sci.* 43 1839–1847.

[B15] Hiebert C. W. Thomas J. B. Somers D. J. McCallum B. D. Fox S. L. (2007). Microsatellite mapping of adult-plant leaf rust resistance gene Lr22a in wheat. *Theor. Appl. Genet.* 115 877–884. 10.1007/s00122-007-0604-3 17646964

[B16] Hovmøller M. S. Walter S. Justesen A. F. (2010). Escalating threat of wheat rusts. *Science* 369–369. 10.1126/science.1194925 20651122

[B17] Huang L. Brooks S. A. Li W. Fellers J. P. Trick H. N. Gill B. S. (2003). Map-based cloning of leaf rust resistance gene Lr21 from the large and polyploid genome of bread wheat. *Genetics* 164 655–664. 10.1017/S0016672303006207 12807786 PMC1462593

[B18] Huerta-Espino J. Singh R. P. German S. McCallum B. D. Park R. F. Chen W. Q. (2011). Global status of wheat leaf rust caused by *Puccinia triticina*. *Euphytica* 179 143–160. 10.1007/s10681-011-0361-x

[B19] Jost M. Singh D. Lagudah E. Park R. F. Dracatos P. (2020). Fine mapping of leaf rust resistance gene Rph13 from wild barley. *Theor. Appl. Genet.* 133 1887–1895. 10.1007/s00122-020-03564-6 32123957

[B20] Keller B. Krattinger S. G. Yahiaoui N. Brunner S. Kaur N. Cloutre C. (2008). “Molecular analysis of fungal disease resistance in wheat,” in *Proceedings of the 11th international wheat genetics symposium*, vol 1, eds Appels R. Eastwood R. Lagudah E. Langridge P. Mackay M. (Sydney, NSW: Sydney University Press Lynne McIntyre and Peter Sharp).

[B21] Knott D. R. (ed.) (1989). “The wheat rust pathogens,” in *The Wheat Rusts — Breeding for Resistance. Monographs on Theoretical and Applied Genetics*, vol 12. (Berlin: Springer).

[B22] Kumar S. Bhardwaj S. Gangwar O. Sharma A. Qureshi N. Kumaran V. (2021). Lr80: a new and widely effective source of leaf rust resistance of wheat for enhancing diversity of resistance among modern cultivars. *Theor. Appl. Genet.* 134 849–858. 10.1007/s00122-020-03735-5 33388887

[B23] Li J. Yang J. Li Y. Ma L. (2020). Current strategies and advances in wheat biology. *Crop J.* 8 879–891. 10.1016/j.cj.2020.03.004

[B24] Marais G. F. Pretorius Z. A. Wellings C. R. McCallum B. Marais A. S. (2005). Leaf rust and stripe rust resistance genes transferred to common wheat from *Triticum dicoccoides*. *Euphytica* 143 115–123. 10.1007/s10681-005-2911-6

[B25] Maroof M. S. Biyashev R. M. Yang G. P. Zhang Q. Allard R. W. (1994). Extraordinarily polymorphic microsatellite DNA in barley: species diversity, chromosomal locations, and population dynamics. *Proc. Natl. Acad. Sci. U. S. A.* 91 5466–5470. 10.1073/pnas.91.12.5466 8202509 PMC44016

[B26] McGrann G. R. Smith P. H. Burt C. Mateos G. R. Chama T. N. MacCormack R. (2014). Genomic and genetic analysis of the wheat race-specific yellow rust resistance gene Yr5. *J. Plant Sci. Mol. Breed.* 3:2. 10.7243/2050-2389-3-2

[B27] McIntosh R. A. Wellings C. R. Park R. F. (1995). *Wheat Rusts: An Atlas of Resistance Genes.* Clayton, VIC: CSIRO publishing.

[B28] McIntosh R. A. Yamazaki Y. Dubcovsky J. Rogers W. J. Morris C. F. Sommers D. J. (2008). “Catalogue of gene symbols for wheat: 2008,” in *Proceedings of the 11th International Wheat Genetics*, eds Appels R. Eastwood R. Lagudah E. Langridge P. Mackay M. McIntyre L. (Sydney, NSW: Sydney University Press).

[B29] McMullen M. Markell S. G. Rasmussen J. B. (2008). *Rust Diseases of Wheat in North Dakota.* Fargo: North Dakota State University Extension Bulletin, 1361.

[B30] Md Hatta M. A. Ghosh S. Athiyannan N. Richardson T. Steuernagel B. Yu G. (2020). Extensive genetic variation at the Sr22 wheat stem rust resistance gene locus in the grasses revealed through evolutionary genomics and functional analyses. *Mol. Plant Microbe Interact.* 33 1286–1298. 10.1094/MPMI-01-20-0018-R 32779520

[B31] Mohler V. Singh D. Singrün C. Park R. F. (2012). Characterization and mapping of Lr65 in spelt wheat ‘Altgold Rotkorn’. *Plant Breed.* 131 252–257. 10.1111/j.1439-0523.2011.01934.x

[B32] Naik B. K. Sharma J. B. Sivasamy M. Prabhu K. V. Tomar R. S. Tomar S. M. S. (2015). Molecular mapping and validation of the microsatellite markers linked to the Secale cereale-derived leaf rust resistance gene Lr45 in wheat. *Mol. Breed.* 35:61. 10.1007/s11032-015-0234-4

[B33] Narang D. Kaur S. Steuernagel B. Ghosh S. Dhillon R. Bansal M. (2019). Fine mapping of Aegilops peregrina co-segregating leaf and stripe rust resistance genes to distal-most end of 5DS. *Theor. Appl. Genet.* 132 1473–1485. 10.1007/s00122-019-03293-5 30706082

[B34] Paux E. Sourdille P. Mackay I. Feuillet C. (2012). Sequence-based marker development in wheat: advances and applications to breeding. *Biotechnol. Adv.* 30 1071–1088. 10.1016/j.biotechadv.2011.09.015 21989506

[B35] Periyannan S. Bansal U. Bariana H. Deal K. Luo M. C. Dvorak J. (2014). Identification of a robust molecular marker for the detection of the stem rust resistance gene Sr45 in common wheat. *Theor. Appl. Genet.* 127 947–955. 10.1007/s00122-014-2270-6 24469473

[B36] Prasad P. Savadi S. Bhardwaj S. C. Gupta P. K. (2020). The progress of leaf rust research in wheat. *Fungal Biol.* 124 537–550. 32448445 10.1016/j.funbio.2020.02.013

[B37] Qiu J. W. Schürch A. C. Yahiaoui N. Dong L. L. Fan H. J. Zhang Z. J. (2007). Physical mapping and identification of a candidate for the leaf rust resistance gene Lr1 of wheat. *Theor. Appl. Genet.* 115 159–168. 10.1007/s00122-007-0551-z 17479240

[B38] Qureshi N. Bariana H. Kumran V. V. Muruga S. Forrest K. L. Hayden M. J. (2018). A new leaf rust resistance gene Lr79 mapped in chromosome 3BL from the durum wheat landrace Aus26582. *Theor. Appl. Genet.* 131 1091–1098. 10.1007/s00122-018-3060-3 29396589

[B39] Rani K. Raghu B. R. Jha S. K. Agarwal P. Mallick N. Niranjana M. (2020). A novel leaf rust resistance gene introgressed from Aegilops markgrafii maps on chromosome arm 2AS of wheat. *Theor. Appl. Genet.* 133 2685–2694. 10.1007/s00122-020-03625-w 32507913

[B40] Raupp W. J. Brown-Guedira G. L. Gill B. S. (2001). Cytogenetic and molecular mapping of the leaf rust resistance gene Lr39 in wheat. *Theor. Appl. Genet.* 102 347–352. 10.1007/s001220051652

[B41] Roelfs A. P. Singh R. P. Saari E. E. (1992). *Rust Diseases of Wheat: Concepts and Methods of Disease Management*. Mexico: International Maize and Wheat Improvement Center (CIMMYT), 1–18

[B42] Ruibal-Mendieta N. L. Delacroix D. L. Meurens M. (2002). A comparative analysis of free, bound and total lipid content on spelt and winter wheat wholemeal. *J. Cereal Sci.* 35 337–342. 10.1006/jcrs.2001.0434

[B43] Saintenac C. Zhang W. Salcedo A. Rouse M. N. Trick H. N. Akhunov E. (2013). Identification of wheat gene Sr35 that confers resistance to Ug99 stem rust race group. *Science* 341 783–786. 10.1126/science.1239022 23811222 PMC4748951

[B44] Saur I. M. Bauer S. Lu X. Schulze-Lefert P. (2019). A cell death assay in barley and wheat protoplasts for identification and validation of matching pathogen AVR effector and plant NLR immune receptors. *Plant Methods* 15:118. 10.1186/s13007-019-0502-0 31666804 PMC6813131

[B45] Singh A. K. Sharma J. B. Singh P. K. Singh A. Mallick N. (2017). Genetics and mapping of a new leaf rust resistance gene in Triticum aestivum L.× Triticum timopheevii Zhuk. derivative ‘Selection G12’. *J. Genet.* 96 291–297. 10.1007/s12041-017-0760-4 28674228

[B46] Singh D. Mohler V. Park R. F. (2013). Discovery, characterisation and mapping of wheat leaf rust resistance gene Lr71. *Euphytica* 190 131–136. 10.1007/s10681-012-0786-x

[B47] Singh D. Park R. F. Bariana H. S. McIntosh R. A. (2001). Cytogenetic studies in wheat XIX. Chromosome location and linkage studies of a gene for leaf rust resistance in the Australian cultivar ‘Harrier’. *Plant Breed.* 120 7–12.

[B48] Soliman A. S. Heyne E. G. Johnston C. O. (1964). Genetic analysis for leaf rust resistance in the eight differential varieties of wheat 1. *Crop Sci.* 4 246–248.

[B49] Stakman E. C. Stewart D. M. Loegering W. Q. (1962). *Identification of Physiologic Races of Puccinia Graminis var. tritici.* Washington, DC: USDA.

[B50] Sun Q. Wei Y. Ni Z. Xie C. Yang T. (2002). Microsatellite marker for yellow rust resistance gene Yr5 in wheat introgressed from spelt wheat. *Plant Breed.* 121 539–541. 10.1046/j.1439-0523.2002.00754.x

[B51] Thomas J. Nilmalgoda S. Hiebert C. McCallum B. Humphreys G. DePauw R. (2010). Genetic markers and leaf rust resistance of the wheat gene Lr32. *Crop Sci.* 50 2310–2317. 10.2135/cropsci2010.02.0065

[B52] Van der Auwera G. A. Carneiro M. O. Hartl C. Poplin R. Del Angel G. Levy-Moonshine A. (2013). From FastQ data to high-confidence variant calls: the genome analysis toolkit best practices pipeline. *Curr. Protoc. Bioinformatics* 43 11–10. 25431634 10.1002/0471250953.bi1110s43 PMC4243306

[B53] Walkowiak S. Gao L. Monat C. Haberer G. Kassa M. T. Brinton J. (2020). Multiple wheat genomes reveal global variation in modern breeding. *Nature* 588 277–283. 10.1038/s41586-020-2961-x 33239791 PMC7759465

[B54] Wang Y. Peng H. Liu G. Xie C. Ni Z. Yang T. (2010). Identification and molecular mapping of a leaf rust resistance gene in spelt wheat landrace Altgold. *Euphytica* 174 371–375. 10.1007/s10681-010-0134-y

[B55] Würschum T. Leiser W. L. Longin C. F. H. (2017). Molecular genetic characterization and association mapping in spelt wheat. *Plant Breed.* 136 214–223. 10.1111/pbr.12462

[B56] Xie Q. Mayes S. Sparkes D. L. (2015). Spelt as a genetic resource for yield component improvement in bread wheat. *Crop Sci.* 55 2753–2765. 10.2135/cropsci2014.12.0842

[B57] Yan G. Chen X. Line R. Wellings C. (2003). Resistance gene-analog polymorphism markers co-segregating with the Yr5 gene for resistance to wheat stripe rust. *Theor. Appl. Genet.* 106 636–643. 10.1007/s00122-002-1109-8 12595992

[B58] Yuan C. Wu J. Yan B. Hao Q. Zhang C. Lyu B. (2018). Remapping of the stripe rust resistance gene Yr10 in common wheat. *Theor. Appl. Genet.* 131 1253–1262. 10.1007/s00122-018-3075-9 29476226

[B59] Zhang N. Yang W. X. Yan H. F. Liu D. Q. Dong C. H. U. Meng Q. F. (2006). Molecular markers for leaf rust resistance gene Lr45 in wheat based on AFLP analysis. *Agric. Sci. China* 5 938–943.

[B60] Zhao F. J. Su Y. H. Dunham S. J. Rakszegi M. Bedo Z. McGrath S. P. (2009). Variation in mineral micronutrient concentrations in grain of wheat lines of diverse origin. *J. Cereal Sci.* 49 290–295. 10.1016/j.jcs.2008.11.007

