# Supplementary material for: Fine Mapping of the Leaf Rust Resistance Gene Lr65 in Spelt Wheat ‘Altgold’
Source: Front Plant Sci. 2021 Jun 28;12:666921. doi: 10.3389/fpls.2021.666921 (PMC8274547; doi:10.3389/fpls.2021.666921)
Supplement: Supplementary file 1 [file Data_Sheet_1.PDF]

Fine mapping of the leaf rust resistance gene *Lr65* in spelt wheat 'Altgold'

Qiang Zhang[1] , Wenxin Wei[1] , Xiangxi Zuansun[1] , Shengnan Zhang[1] , Chen Wang[1] , NannanLiu[1], Lina Qiu[1],

Weidong Wang[1], Weilong Guo[1],Jun Ma[1], Huiru Peng[1], Zhaorong Hu[1], Qixin Sun[1], Chaojie Xie[1*]

[1]State Key Laboratory for Agrobiotechnology, Key Laboratory of Crop Heterosis and Utilization (MOE), Key Laboratory of Crop Genetic Improvement, College of Agronomy and Biotechnology, China Agricultural University, Beijing, China.

** Corresponding author:*

xiecj127@126.com (86-010-62731064)

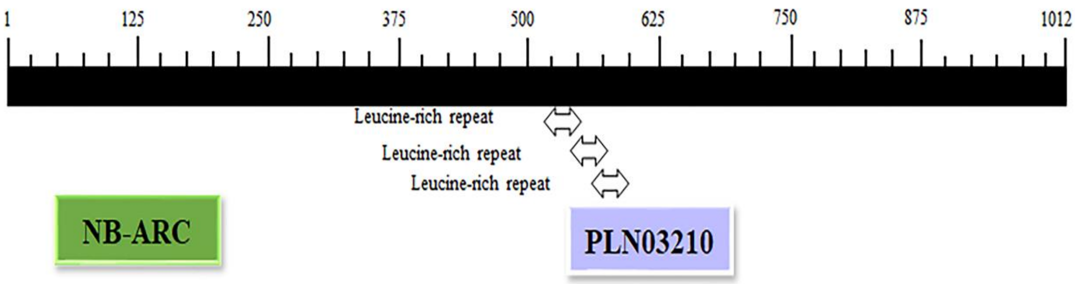

Fig. S1 Structural prediction of amino acids encoded by *TraesCS2A02G001500* gene . NB-ARC is a loci capable of binding to nucleotides. PLN03210is a multiple leucine-rich repeat sequence.

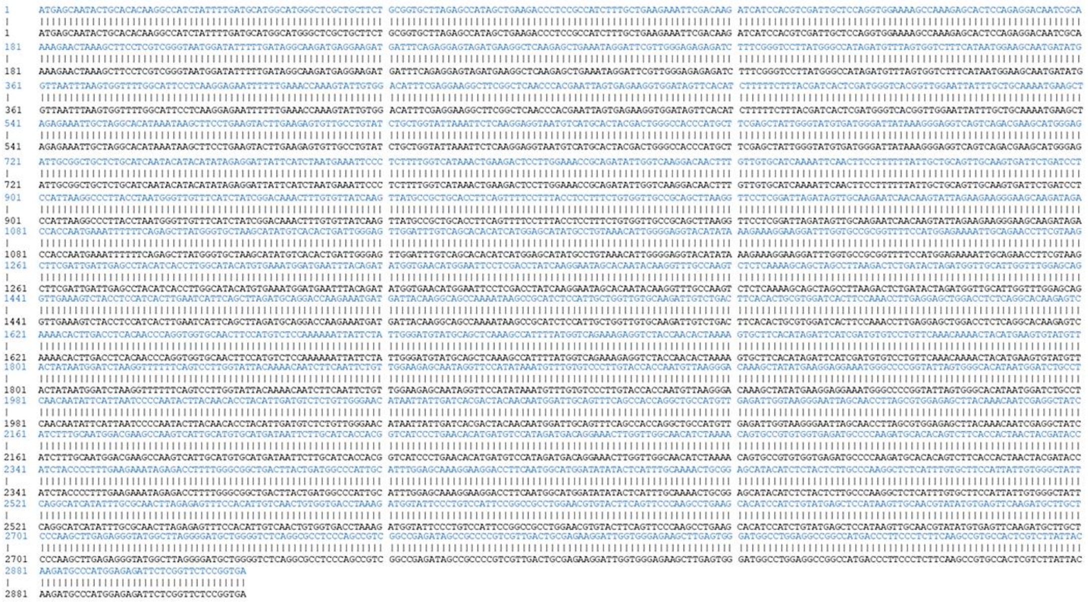

Fig. S2 Nucleotide sequence of the annotated gene *TraesCS2A02G001400* compared between resistant (Altgold) and susceptible (CS) parents. Blue and black represent Altgold and CS, resp.

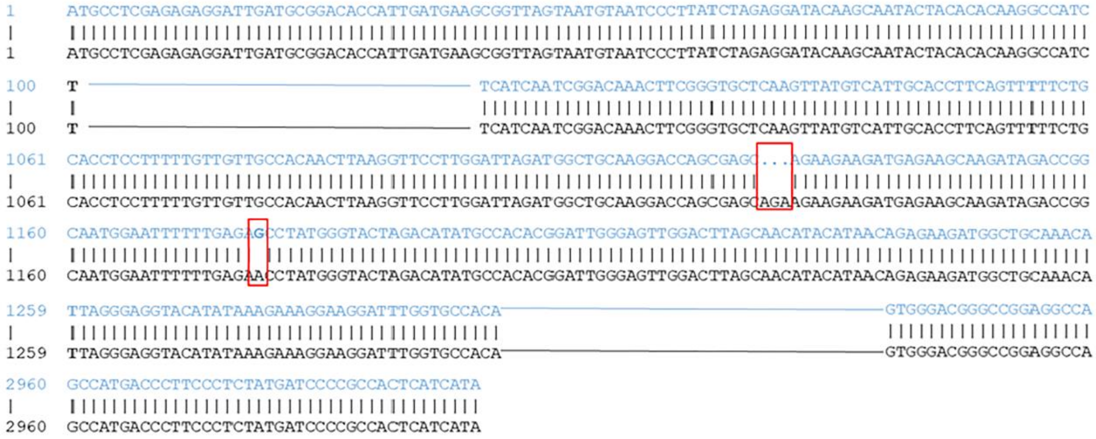

FigS3. Nucleotide sequence of the annotated gene *TraesCS2A02G001500* compared between resistant (Altgold) and susceptible (CS) parents. Blue and black represent Altgold and CS, resp. The horizontal line represents the omission of part of the same sequence of both parents. The red box shows a 3-bp indel and a SNP.

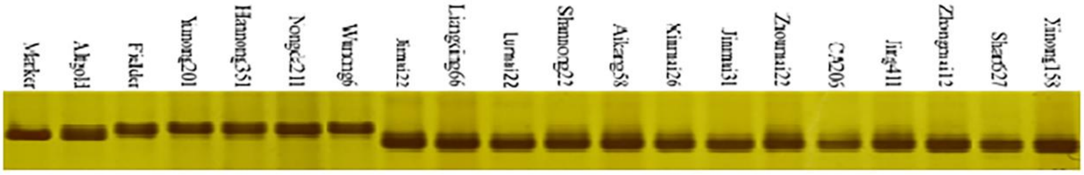

Fig. S4 PCR products of Altgold and 18 Chinese cultivars

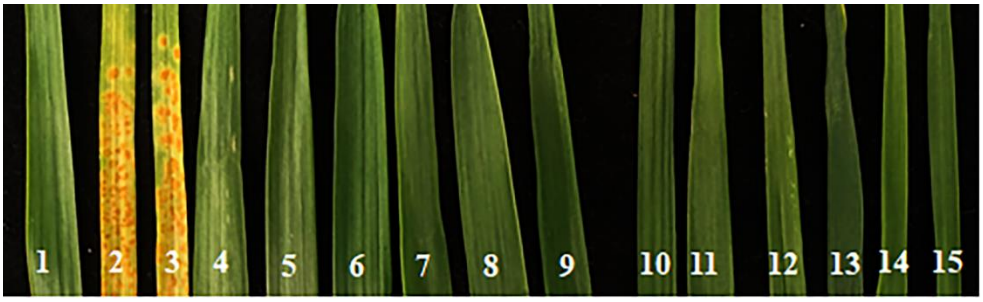

Fig. S5 Images of plants in the greenhouse.1-3 are Xuexao / Altgold disease resistant progeny, Shiyou20 and Zhongmai1062, respectively; 4-9 are backcross progeny of Xuezao / Altgold disease resistant progeny and Shiyou20; 10-15 are backcross progeny of Xuezao / Altgold disease resistant progeny and Zhongmai1062.
